# Consumption of fruits and vegetables among Peruvian adults: Analysis of a national health survey 2017–2018

**DOI:** 10.1371/journal.pgph.0004222

**Published:** 2025-03-13

**Authors:** Wilmer Cristobal Guzman-Vilca, Rodrigo M. Carrillo-Larco, Carla Tarazona-Meza

**Affiliations:** 1 CRONICAS Centre of Excellence in Chronic Diseases, Universidad Peruana Cayetano Heredia, Lima, Peru; 2 Hubert Department of Global Health, Rollins School of Public Health, Emory University, Atlanta, Georgia, United States of America; 3 Emory Global Diabetes Research Center, Emory University, Atlanta, Georgia, United States of America; 4 Program in Human Nutrition, Department of International Health, Bloomberg School of Public Health, Johns Hopkins University, Baltimore, Maryland, United States of America; 5 Nutrition and Dietetics, Universidad Científica del Sur, Lima, Perú; Indian Council of Medical Research, INDIA

## Abstract

The WHO recommends consuming ≥400 g/day of fruits and vegetables daily as part of a healthy diet to prevent non-communicable diseases (NCDs). Data on fruits and vegetables intake is scarce in several countries, including Peru. However, it remains crucial to monitor policies to reduce the burden of non-communicable diseases. Cross-sectional analysis of a representative at the national level survey of Peruvian adults conducted in 2017–2018. Consumption of fruits and vegetables, stratified by socio-demographic and health-related variables, was estimated from a single 24-hour dietary recall questionnaire. Regression models were conducted to assess the potential association with low consumption of fruits and vegetables. The mean consumption of fruits and vegetables was 153.8 (95% CI: 133.4–174.2) g/day. Only 13.6% of the population consumed ≥400 g/day of fruits and vegetables. People with obesity (120 g/day), unaware diabetes (79.6 g/day) and unaware hypertension (51.1 g/day) had the lowest mean consumption. A consumption of <400 g/day was associated with obesity (OR): 2.56 (95% CI: 1.22–5.37) and having hypertension (OR: 3.32 (95% CI: 1.16–9.5)). Only 14 out of 100 Peruvian adults consume the recommended daily amount of fruits and vegetables and the mean consumption of fruits and vegetables is less than 2 portions a day. There is an urgent need for multi-sectoral health policies focused on increasing the access and consumption of fruits and vegetables as part of a healthy diet to reduce the burden of NCDs.

## Introduction

Non-communicable diseases (NCDs) are responsible for ~40 millions of all deaths worldwide [1,2]. Different risk factors contribute to the burden of NCDs, including modifiable dietary risk factors such as low consumption of fruits and vegetables [3]. The World Health Organization (WHO) recommends consuming at least 400 grams (~5 portions) of fruits and vegetables per day, as part of a healthy diet to prevent NCDs [4]. Nonetheless, up-to-date precise estimates on the consumption levels of fruits and vegetables are scarce in countries from Latin America. This information is crucial to monitor and evaluate the adherence to dietary guidelines recommendations, and to inform dietary policies, to ultimately reduce the prevalence of NCDs.

Global and local studies have assessed fruits and vegetables consumption in Peru [5–8]. One global study reported that the mean consumption of fruits and vegetables in Peru was below the recommendations, 331 g/day in 2010 [8]. Even though informative, estimates for Peru were modelled using the Bayesian model approach, and did not leverage on national data. Another study analyzed information from the demographic health survey (ENDES in Spanish) in Peru which found that only 11% of adults (18+ years) in Peru consumed ≥400 grams/day of fruits and vegetables in 2019 [5]. These estimates were based on self-reported data about eating habits in the previous week [5]. Similarly, other epidemiological studies in Peru have reported fruits and vegetables consumption estimates in specific populations [6,7]. Nevertheless, these results were based on questionnaires which may be more susceptible to measurement error on estimating the precise amounts intake, compared with 24-hour recalls. Dietary 24-hour recalls usually provide more accurate estimates of food consumption, [3,9] which could be useful in low-resource settings when further guidance is needed to estimate the portion sizes and intake frequency.

We aimed to quantify the consumption of fruits and vegetables in Peruvian adults from a nationally-representative dietary intake data in Peru. We also described the associated factors to consume less than 400 grams per day of both, fruits and vegetables.

## Methods

### Study design and population

Cross-sectional analysis of Peruvian adults who participated in the National Survey of Food and Nutritional Surveillance by Life Stages 2017–2018 (VIANEV by its acronyms in Spanish) conducted by the Peruvian National Centre for Food and Nutrition (CENAN, for its acronym in Spanish) [10]. This survey was conducted in 2017–2018 on a nationally representative sample of Peruvian adults [10]. This is the most recent survey in Peru that included biomarkers, along with a dietary intake assessment, and physical measurements (blood pressure, height, weight and waist circumference (WC). This survey adhered to ethical guidelines and followed a standard protocol for all measurements [10].

The VIANEV survey was based on a subsample of the Peruvian National Household Survey 2017 (ENAHO by its acronyms in Spanish) and included adults aged 18–59 years old living in both urban and rural areas. The VIANEV survey was designed to be representative at the national level and to three strata: 1) Lima, 2) Urban areas except Lima, and 3) Rural areas [10]. To calculate the survey sample size in each strata, the formula presented in S1 Fig was used and a two-stage probability sampling design was followed [10]. First, clusters were randomly selected considering the three strata. Then, within each cluster, households were randomly selected. To be selected for the survey, participants had to meet the following inclusion criteria: 1) adults aged 18–59 years and 2) fasting 9–12 hours for blood biomarkers. The following participants were excluded: 1) pregnant and postpartum women, 2) adults with medication that could alter glucose and lipid profiles, and 3) adults with congenital diseases that could alter their anthropometric measurements (e.g., Down syndrome) [10].

We included all adults with complete data on dietary intake and ponderation data. No further exclusion criteria were applied. A flowchart of the data cleaning process is presented in S2 Fig.

### Dietary intake measurement

Participants completed one or two 24-hour recalls administered by professional nutritionists familiar with local dietary patterns previously trained on interview techniques and standardized [10]. Participants were asked to report all foods and beverages consumed in the last 24 hours prior the interview using the multi-pass standardized methodology. When possible, food items and standard portion sizes were directly weighted using a foods scale and standardized booklets with portion sizes and to-scale pictures of common food items to determine the quantity (in grams or milliliters) of each food and beverage consumed. The day in which the 24-hour recall took place was randomly selected to guarantee all days of the week were included. As all participants underwent at least one 24-hour recall and only one random subsample (20% of participants) underwent two non-consecutive 24-hour recalls, only the data from the first 24-hour recall was used for this study. To further support this decision, we compared the mean consumption of fruits and vegetables between the first and second 24-hour recall amongst those with two 24-hour recalls, using paired t-tests. There was no statistically significant difference in the mean consumption of fruits and vegetables (*p* = 0.1886) between the two 24-hour recalls.

### Fruit and vegetable consumption estimation

The outcome variables were the consumption in gram units of: i) fruits, ii) vegetables, and iii) fruits and vegetables combined at the individual level per day. The total daily consumption was estimated adding up the food items classified as fruits and vegetables per participant per day. Consistent with previous evidence, [3] we included only fresh fruits and excluded fruits desserts and fruit juices as they regularly have added sugar [3]. We included raw, salad, cooked and soup vegetables. Similarly to previous studies, [11,12] we estimated that one portion of vegetable soup (250 g) was considered to include 80 g of soup vegetables. This was also supported by a recent study that showed that one portion (250 g) of mixed dried vegetables soup contains approximately 84 g/day of fresh vegetable content [13].

To estimate the daily consumption of fruits and vegetables, we added the intake amount in grams of fruits, vegetables, and fruits and vegetables combined in a single 24-hour recall for each individual (i.e., total consumption in grams).

### Sociodemographic and health variables

The outcome variables were stratified by socio-demographic and health-related variables. Socio-demographic variables included were sex, age groups, urban-rural location, and educational level. Health-related variables included were body mass index category, abdominal obesity, diabetes, and hypertension.

Participants education level was reported based on their highest reported schooling: no or less than primary education, primary education, secondary education, higher education, and post-graduate education. For this study, we categorized education into three groups: low (no or less than primary/primary education), medium (secondary education), and high (higher/post-graduate education).

BMI (kg/m2) was calculated using measured weight and height. For descriptive purposes, BMI was divided into three categories: normal (BMI 18-5-24.9 kg/m2), overweight (BMI 25–29.9 kg/m2), and obesity (BMI ≥30 kg/m2). Those with underweight (BMI <18.5 kg/m2) were not described in our results as they only represented 0.06% of the sample.

Abdominal obesity was determined using the waist circumference (WC) measurement. Individuals were grouped into two categories based on WC: no abdominal obesity (WC < 90 cm in men and < 80 cm in women) and abdominal obesity (WC ≥ 90 cm in men and ≥ 80 cm in women [14].

Although the survey encompassed three blood pressure measurements, the third reading was taken in less than 2% of participants. Therefore, we solely utilized the second blood pressure measurement, discarding the first and third records [15,16].

Hypertension was defined as having a blood pressure measurement equal to or greater than 140/90 mmHg, or answering affirmatively to either, the question about self-reported hypertension diagnosis or the query about receiving prescribed antihypertensive medication. Individuals who acknowledged being diagnosed with hypertension or receiving antihypertensive treatment were classified as aware of their hypertension status. Those with blood pressure meeting the hypertensive threshold but denying a diagnosis or treatment were categorized as unaware of their condition. The otherwise healthy group, without hypertension, comprised individuals with blood pressure below 140/90 mmHg who also denied previous diagnosis or treatment for hypertension.

Type 2 diabetes mellitus (T2DM) status was determined by measuring fasting plasma glucose levels and self-reported information on diabetes diagnosis and treatment. Individuals with diabetes were identified as having fasting plasma glucose levels equal to or greater than 126 mg/dL or answering positively to either the question about self-reported diabetes diagnosis or the inquiry about receiving prescribed diabetes medication. Those who self-reported a diabetes diagnosis or treatment were considered aware of their diabetes status. Individuals with fasting plasma glucose levels meeting the diabetic threshold but denying diagnosis or treatment were classified as unaware of their condition. The otherwise healthy group, without diabetes, included individuals with fasting plasma glucose levels below 126 mg/dL who also denied diagnosis or treatment of diabetes.

### Statistical analysis

Individuals were classified according to whether they met the recommended daily consumption of fruits and vegetables or not (≥400 g/day; no/yes). The daily consumption of fruits and vegetables at the population level is presented as mean consumption estimates along with their 95% confidence interval (95% CI). To describe the proportion of the population that met the recommended daily consumption of fruits and vegetables, we used prevalence estimates along with their 95% CI.

The characteristics of the population were described accounting for the complex survey design. To describe which variables were associated with a consumption of less than 400 g/day of fruits and vegetables, we fitted a logistic regression model using the *svyglm* command in R. A p <0.05 was considered statistically significant. The regression model accounted for the complex survey design, and it was adjusted for sex, age, urban/rural location, educational level, BMI category, abdominal obesity, diabetes, and hypertension status. All analyses were conducted in R (version 4.0.3, R Core Team, Vienna, Austria). The analysis code and datasets are also available as Supplementary Material (S1 and S2 Code).

### Ethics

This study used anonymized nationally-representative survey data that are in the public domain (https://www.datosabiertos.gob.pe/). Our study involves a secondary-data analysis and no human subjects were directly involved in the project, entailing thus a minimal risk. Thus, we did not seek approval by an Institutional Review Board.

### Role of the funding source

The funder had no role in the study design, analysis, interpretation, or decision to publish. The authors are collectively responsible for the accuracy of the data. The arguments and opinions in this work are those of the authors alone, and do not represent the position of the institutions to which they belong.

## Results

For this study, 913 people were included. The proportion of men was 42% and the mean age was 37.7 years. The prevalence of overweight and obesity were 37.2% and 27.3%, respectively. People with diabetes represented 14.0% and those who knew their diagnosis were 4.6%. People with total (both diagnosed and undiagnosed) hypertension were 9.9% and those with self-reported hypertension were 7.1% (Table 1).

**Table 1:** Weighted distribution of demographic and clinical variables in the study population, overall and by sex.

Characteristics	Total n=913	Men n=386	Women n=527
**Age (mean and 95% CI, years)**	37.7 (36.9–38.6)	37.1 (35.7–38.5)	38.2 (37.1–39.3)
**Proportion of people aged 18–29 years (95% CI, %)**	29.5 (26.4–32.9)	33.2 (27.9–39.1)	26.9 (22.8–31.4)
**Proportion of people aged 30–39 years (95% CI, %)**	26.2 (22.9–29.8)	25 (20.4–30.3)	27.1 (22.8–31.8)
**Proportion of people aged 40–49 years (95% CI, %)**	24.3 (21.2–27.7)	20.5 (16.5–25.1)	27.1 (22.8–31.8)
**Proportion of people aged 50–59 years (95% CI, %)**	19.9 (17.1–23.1)	21.3 (17.1–26.3)	18.9 (15.5–22.9)
**Body mass index (mean and 95% CI, kg/m^2^)**	27.4 (27–27.8)	26.7 (26.1–27.3)	27.9 (27.4–28.5)
**Proportion of people with normal weight (95% CI, %)**	34.8 (31.4–38.3)	38.9 (33.3–44.7)	31.8 (27.4–36.7)
**Proportion of people with overweight (95% CI, %)**	37.2 (33.5–41.2)	37.8 (32.3–43.6)	36.9 (32.1–42.0)
**Proportion of people with obesity (95% CI, %)**	27.3 (24.0–30.9)	22.7 (18.1–28.2)	30.6 (26.0–35.6)
**Waist circumference (mean and 95% CI, cm)**	92 (91–92.9)	92.7 (91.1–94.3)	91.4 (90.2–92.6)
**Proportion of people with central obesity (95% CI, %)**	71.4 (67.9–74.7)	57 (51.3–62.5)	82.4 (78.4–85.8)
**Proportion of people with diabetes (95% CI, %)**	14 (11.4–17.1)	15.1 (11.2–20.1)	13.2 (10–17.2)
**Proportion of people with self-reported diabetes (95% CI, %)**	4.6 (3.2–6.5)	3.7 (2–6.9)	5.2 (3.4–7.8)
**Proportion of people with unaware diabetes (95% CI, %)**	8.9 (6.8–11.4)	11 (7.8–15.3)	7.3 (5–10.6)
**Proportion of people with hypertension (95% CI, %)**	9.9 (7.8–12.4)	12.2 (8.9–16.5)	8.1 (6–11)
**Proportion of people with self-reported hypertension (95% CI, %)**	7.1 (5.4–9.4)	8.2 (5.5–12)	6.4 (4.5–9)
**Proportion of people with unaware hypertension (95% CI, %)**	2.5 (1.6–3.9)	3.8 (2.1–6.8)	1.4 (0.7–2.8)

The mean consumption was 96.3 (95% CI: 82.2–110.4) g/day for fruits and 57.5 (95% CI: 44.6–70.3) g/day for vegetables. For both, fruits and vegetables combined, the mean consumption was 153.8 (95% CI: 133.4–174.2) g/day. The 13.6% (95% CI: 11.2%–16.4%) of the population consumed ≥400 g/day of fruits and vegetables.

### Estimates of fruits and vegetables consumption according to sex and 10-year age groups

The mean consumption of fruits and vegetables was slightly higher in woman (166.5 g/day) than in men (136.3 g/day) mainly due to a higher fruit consumption in woman than men (109.7 g/day vs 77.8 g/day) (Table 2). We observed the same profile for the prevalence of recommended consumption of ≥400 g/day (Table 2). Stratified by age groups, mean consumption of fruits and vegetables was slightly higher in those aged 50–59 years compared to younger age groups, but there were no age-specific patterns in both mean consumption and prevalence of consumption of ≥400 g/day.

**Table 2:** Fruits and vegetables consumption by socio-demographic and health-related variables.

	Total fruit consumption			Total vegetable consumption		Total fruit and vegetable consumption			
**Variable**		**Mean and 95% CI (g/day)**	**p value**	**Mean and 95% CI (g/day)**	**p value**	**Mean and 95% CI (g/day)**	**p value**	**Prevalence of consumption ** **of ≥400** ** g/day (% and 95% CI)**	**p value**
**Sex**	Men	77.8 (58.9–96.8)	0.05	58.4 (35.7–81.1)	0.56	136.3 (106.9–165.6)	0.31	11.9 (8.8–15.9)	0.49
	Women	109.7 (89.8–129.7)		56.8 (40.6–73)		166.5 (139.9–193.1)		14.8 (11.7–18.6)	
**Age groups**	18–29	77.6 (54.2–101)	0.38	70.3 (42.4–98.2)	0.06	147.9 (110–185.8)	0.49	14.9 (10.7–20.4)	
	30–39	104.8 (75.3–134.2)		45.5 (25.2–65.7)		150.2 (114.4–186)		13.6 (9.6–19.1)	0.76
	40–49	108.9 (80.1–137.6)		37.9 (23–52.8)		146.8 (113.3–180.2)		11.1 (7.2–16.7)	
	50–59	97.8 (69.1–126.5)		78.2 (39.1–117.3)		175.9 (129–222.8)		14.6 (9.9–21.1)	
**Area of residence**	Rural	107.1 (79.9–134.3)	0.17	59.3 (35.8–82.8)	0.38	166.4 (131.5–201.2)	0.10	15.1 (11–20.3)	0.18
	Urban	93.4 (77.1–109.8)		57 (41.9–72)		150.4 (126.3–174.6)		13.2 (10.4–16.6)	
**Educational level**	High	104.5 (82.5–126.5)	0.75	58.6 (37.5–79.7)	0.37	163.1 (132.1–194.1)	0.62	13.6 (10.1–18.2)	0.68
	Low	89 (62.4–115.6)		37.6 (19.6–55.7)		126.6 (95.6–157.7)		11.1 (7.4–16.5)	
	Medium	90 (66.8–113.2)		66.6 (42.8–90.4)		156.6 (121.6–191.6)		14.9 (11–19.8)	
**Body mass index category**	Normal	98.3 (72.4–124.2)	0.18	69.2 (42.8–95.7)	0.08	167.5 (130.7–204.4)	<0.05	15.3 (11.3–20.3)	<0.05
	Obesity	82.8 (57.4–108.2)		37.2 (18.9–55.6)		120 (87.9–152.1)		15.9 (12–20.7)	
	Overweight	101.2 (79.8–122.6)		60.6 (39.3–82)		161.8 (131.1–192.5)		7.8 (4.8–12.6)	
**Abdominal obesity**	No	76.6 (56.2–97.1)	0.42	73.6 (42.9–104.3)	0.27	150.2 (113.7–186.8)	0.79	13.3 (9.2–18.7)	1.00
	Yes	99 (81.6–116.4)		51.9 (37.4–66.4)		150.9 (127.3–174.5)		12.8 (10.1–16.1)	
**Diabetes status**	Not diabetes	98.5 (82.4–114.6)	0.45	57.1 (43.1–71.1)	0.22	155.6 (132.7–178.6)	0.19	13.2 (10.6–16.4)	0.21
	Only self-reported diabetes	107.2 (34.4–179.9)		90.7 (8.7–172.7)		197.9 (84.3–311.4)		14.5 (5.8–31.6)	
	Unaware diabetes	62 (27.8–96.1)		17.7 (0–38.1)		79.6 (36.5–122.8)		7.3 (2.9–17.2)	
	Total diabetes	78.5 (43.6–113.4)		44.4 (11–77.8)		122.9 (72.1–173.8)		10 (5.3–17.9)	
**Hypertension status**	Not hypertension	96 (81.1–111)	0.07	62.4 (48.1–76.6)	0.06	158.4 (136–180.8)	<0.05	14.2 (11.6–17.3)	<0.05
	Unaware hypertension	40.6 (0.3–80.8)		10.5 (0–23.4)		51.1 (9–93.2)		2.2 (0.3–16.4)	
	Total hypertension	66.7 (41.1–92.4)		22.6 (0–46.1)		89.3 (52.3–126.4)		2.6 (0.9–7)	
	With self–reported hypertension	75.5 (44.1–106.8)		26.6 (0–57.7)		102.1 (54.9–149.2)		2.7 (0.8–8.5)	

### Estimates of fruits and vegetables consumption according to urban/rural location

The mean consumption of vegetables was similar between people living in urban (57.0 g/day) and rural (59.3 g/day) locations. Also, there were no substantial differences in the mean consumption and prevalence of consumption of ≥400 g/day of fruits and vegetables.

### Estimates of fruits and vegetables consumption according to education level

The mean consumption of fruits and vegetables was slightly lower in people with low education (126.6 g/day) compared with those with high (163.1 g/day) and medium (156.6 g/day) education, mainly because of low vegetable consumption. The same pattern occurred for the prevalence of ≥400 g/day, but differences across educational levels were not significant.

### Estimates of fruits and vegetables consumption according to BMI categories and abdominal obesity

People with obesity (120.0 g/day) had a lower mean consumption of fruits and vegetables compared with those with optimal BMI (167.5 g/day) and overweight (161.8 g/day). This finding was true for both fruit and vegetable consumption. The prevalence of consumption of ≥400 g/day in people with optimal BMI and overweight doubled the prevalence in those with obesity. In contrast, there were virtually no differences in the mean consumption and prevalence of consumption of ≥400 g/day of fruits and vegetables in those with and without abdominal obesity.

### Estimates of fruits and vegetables consumption according to diabetes and hypertension status

People with diabetes (122.9 g/day) had slightly lower consumption of fruits and vegetables compared with those without diabetes (155.6 g/day); this was also true for hypertension (89.3 g/day vs 158.4 g/day in people with and without hypertension, respectively). Among people with diabetes and hypertension, those who knew their diagnosis had a higher consumption of fruits and vegetables than those who were unaware of it. Also, the prevalence of consumption of ≥400 g/day of fruits and vegetables in people with self-reported diabetes doubled the prevalence in people with unaware diabetes.

### Factors associated with consuming <400 g/day of fruits and vegetables

Compared with people with normal BMI, people with obesity were 2.6 times more likely to consume <400 g/day of fruits and vegetables (OR = 2.56, 95% CI: 1.22–5.37) (Table 3). Also, people with hypertension were 3.3 times more likely to consume <400 g/day of fruits and vegetables compared with people without hypertension (OR = 3.32, 95% CI: 1.16–9.50). No other factors such as sex, age, area of residence, educational level, abdominal obesity, and diabetes status were significantly associated with consuming <400 g/day of fruits and vegetables.

**Table 3:** Associated factors with consuming <400 g/day of fruits and vegetables.

Variables	<400 g/day consumption of fruits and vegetables	
	Odds Ratio (95% CI)	p value
**Sex**		
** Men**	Reference	
** Women**	0.88 (0.55–1.4)	0.59
**Age groups**		
** 18–29 years**	Reference	
** 30–39 years**	1.1 (0.6–2)	0.76
** 40–49 years**	1.01 (0.54–1.88)	0.99
** 50–59 years**	0.63 (0.34–1.17)	0.14
**Area of residence**		
** Rural**	Reference	
** Urban**	1.38 (0.84–2.26)	0.21
**Educational level**		
** Low education**	Reference	
** Medium education**	0.69 (0.38–1.25)	0.22
** High education**	0.82 (0.43–1.58)	0.56
**Body mass index category**		
** Normal**	Reference	
** Overweight**	1.27 (0.71–2.26)	0.42
** Obesity**	2.56 (1.22–5.37)	<0.05
**Abdominal obesity**		
** No**	Reference	
** Yes**	0.69 (0.36–1.32)	0.26
**Diabetes status**		
** Not with diabetes**	Reference	
** With diabetes**	1.46 (0.75–2.87)	0.27
**Hypertension status**		
** Not with hypertension**	Reference	
** With hypertension**	3.32 (1.16–9.5)	<0.05

## Discussion

### Main findings

Leveraging on a nationally representative survey of Peruvian adults, we quantified the daily mean consumption of fruits and vegetables, along with the proportion of the population meeting the WHO recommended daily consumption. We also described which factors were linked to consuming less than the recommended daily quantity of fruits and vegetables. We observed that, on average, adults consume less than half (154 g/day) the recommended daily intake of fruits and vegetables while only 14% of adults consumed more than 400 g/day of fruits and vegetables. People with obesity (120 g/day), unaware diabetes (79.6 g/day) and unaware hypertension (51.1 g/day) had the lowest daily mean consumption. People with obesity, unaware diabetes, and hypertension showed the lowest prevalence of the recommended daily consumption of fruits and vegetables. Participants who had obesity and hypertension were more likely to consume less than 400 g/day fruits and vegetables in a daily basis.

### Public health implications

As part of a healthy diet, the WHO recommends consuming at least 5 portions of fruits and vegetables each day to reduce morbidity and mortality from NCDs. Furthermore, transitioning to plant-based diets such as those rich in fruits and vegetables (e.g., Mediterranean diet) have not only a positive impact on human heath but also in planetary health, reducing diet-related greenhouse gas emissions [17–19]. In this study, we have found alarmingly low levels of consumption of fruits and vegetables in Peruvian adults, as their mean daily consumption was less than 2 portions, and less than 14% of the population comply with eating ≥5 portions daily. These findings call for urgent actions from public health organizations at the national and regional level to increase fruits or vegetables intake at the population level. There is a wide spectrum of health interventions that have proven to be effective at increasing the consumption of fruits and vegetables in adults [20]. Food pricing interventions strategies that reduce the price of fruits and vegetables and increase the price of sweets have shown to increase fruits and vegetable consumption, [21] including in LMICs [22]. Technical support for increasing agricultural production, diversification and conservation through national policies is also needed [23]. As well, national governments must ensure that fruits and vegetables are available and physically accessible for all people; for example, mobile markets could be helpful for delivering fruits and vegetables in rural areas [17]. Moreover, mass media intervention strategies targeting nutrition behaviors are effective in increasing fruits and vegetable intake in both adults and young people [24]. Besides, at the individual level, primary care providers (e.g., physicians or nutritionists) could provide counseling interventions to their patients regarding healthy diets including fruits and vegetables [25]. Clinical guidelines from global [26,27] and national organizations [28,29] emphasize the inclusion of fruits and vegetables as part of the nutritional therapy in both people with diabetes and hypertension. As we have found alarmingly low levels of consumption of fruits and vegetables in these populations, counseling on healthy dietary habits should be reinforced to reduce the risk of complications derived from uncontrolled diabetes or hypertension [26,27].

In Peru, there have been different strategies to promote the consumption of fruits and vegetables as part a healthy diet. The Peruvian National Centre for Food and Nutrition (CENAN) developed dietary guidelines based on foods (GABAS acronym in Spanish) for the Peruvian population considering the country’s food diversity, where they highlighted the importance to consume at least 400 grams/day of fruits and vegetables [30] that may be available locally at different seasons. The National Healthy Eating Law passed in 2019 promotes the creation of healthy dining rooms in different public institutions (e.g., hospitals) [31]. Following the recommendations from the CENAN dietary guidelines, these dining rooms could increase the intake of fruits and vegetables in the general population. Similar recommendations, or additional implementation strategies can be applied to social programs, to improve the adherence to national recommendations and improve the fruit and vegetable consumption. Likewise, multi-sectoral health policies at local and national levels can focus on increasing the access of fruits and vegetables throughout the year.

### Research in context

A global study conducted in 28 LMICs used data from nationally-representative surveys reported than only 18% of people residing in these LMICs consumed the recommended amount of fruits and vegetables, and these proportion was even lower for LAC countries (8%) [32]. Our estimates for Peru (13.6%) are higher than those reported for LAC, particularly higher than Brazil (6.1%) and Costa Rica (8.1%), but lower than Chile (16.6%) and Grenada (24.7%) [32]. Potential explanations for these differences include different methods to measure dietary intake (i.e., health questionnaire in the study by Frank et al [32] vs 24-hour recall in our study) and different age ranges (15+ years in the study by Frank et al [32] vs 15–59 years in our study).

In Peru, a recent national study produced estimates of adequate consumption of fruits and vegetables using dietary questionnaires [5]. Participants were asked how many portions of fruits and vegetables per day they have eaten in the last seven days. Their results showed that 10.7% and 11% of people in Peru consumed ≥5 servings/day of fruits and vegetables in 2014 and 2019, respectively [5]. Compared to their results in 2019, ours are slightly higher (13.6% in 2017–2018), which can be attributed to the different methods to measure dietary intake (i.e., health questionnaire vs 24-hour recall). Arguably, the data we leveraged with 24-hour recalls provide much robust evidence. Also, as people aged ≥60 years tend to consume fewer portions of fruits and vegetables; thus, their lower estimates could have been attributed to the fact that they included all adults ≥15 years and we only included adults aged 18–59 years.

Most data in Peru on fruit and vegetable consumption have focused on specific populations or regions. For example, one study in Lima reported that 8.5% of the regular consumers of community kitchens consumed more than 400 g/day of fruits and vegetables [6]. Their lower results compared to ours could be attributed to the fact that community kitchens commonly face economic constraints which difficult the access to fruits and vegetables. Another study in one district of Lima reported that 26.2% of caregivers of children consumed more than 400 g/day of fruits and vegetables [7]. Their higher results compared to ours could be explained by the more advanced socio-economic growth in Lima compared to other regions in Peru, thus a higher average income to pursue healthier food choices.

### Strengths and limitations

We have reported the first estimates of fruits and vegetable consumption based on data from 24-hour recalls at the national level, which allowed us to quantify more precise results than those obtained from semi-quantitative or qualitative dietary questionnaires. We also described the results by several socio-demographic and health-related variables. Nonetheless, we must acknowledge some limitations. First, our estimates were derived from a single 24-hour recall, which does not represent the usual dietary intake of the population and may have underestimated it. As the consumption of fruits and vegetables is low in Peru, data from a single 24-hour recall may not represent the true daily consumption of fruits and vegetables. Also, food reporting during these recalls could have been affected by recall bias, [33] underestimating fruits or vegetables consumption. Future studies reporting dietary estimates at the national level could yield more accurate results if they incorporate 24-hour recalls in two or three non-consecutive days. Second, data were collected almost seven years ago (2017–2018). We argue that, in absence of major dietary reforms and policies, our estimates are still valid today; nonetheless, new data are much needed to monitor these indicators since 2017–2018, to inform policies, and to set goals for the next years. In this line, while COVID-19 may have impacted the overall dietary profiles in the population,[34,35] we hypothesize this effect has disappeared now, so current estimates should be similar to those we reported based on 2017–2018 data. This is largely speculative and warrants verification. Third, other than some broad socio-demographic variables, we did not include information on household income or household food expenditure on fruits and vegetables. Future work should explore how much of the low fruit and vegetable consumption is driven by economic hardship. Fourth, our data were not designed to explore seasonal pattern, [36] which may affect availability and access to fruits and vegetables in Peru. In this line, future work should how much seasonality affects fruits and vegetable consumption in the different country regions; furthermore, whether climate change events disrupt supply chains affecting access and availability of fruits and vegetables.

## Conclusions

Only 14 out of 100 Peruvian adults consumed the recommended daily quantity of fruits and vegetables and the mean consumption of fruits and vegetables is less than 2 portions a day. People with obesity and hypertension are much more likely to consume less than 5 portions of fruits and vegetables a day. There is an urgent need to increase and promote the consumption of fruits and vegetables as a key component of a healthy diet to prevent and reduce the burden of NCDs.

## Supporting information

S1 Fig Formula for calculating the survey sample size. (DOCX)

S2 Fig Flowchart of data cleaning and inclusion criteria. (DOCX)

S1 Code Data extraction. (R)

S2 Code Data analysis. (R)
