# Supplementary material for: Consumption of fruits and vegetables among Peruvian adults: Analysis of a national health survey 2017–2018
Source: PLOS Glob Public Health. 2025 Mar 13;5(3):e0004222. doi: 10.1371/journal.pgph.0004222 (PMC11906043; doi:10.1371/journal.pgph.0004222)
Supplement: S1 Fig — (DOCX) [file pgph.0004222.s001.docx]

# **S1 Fig.**


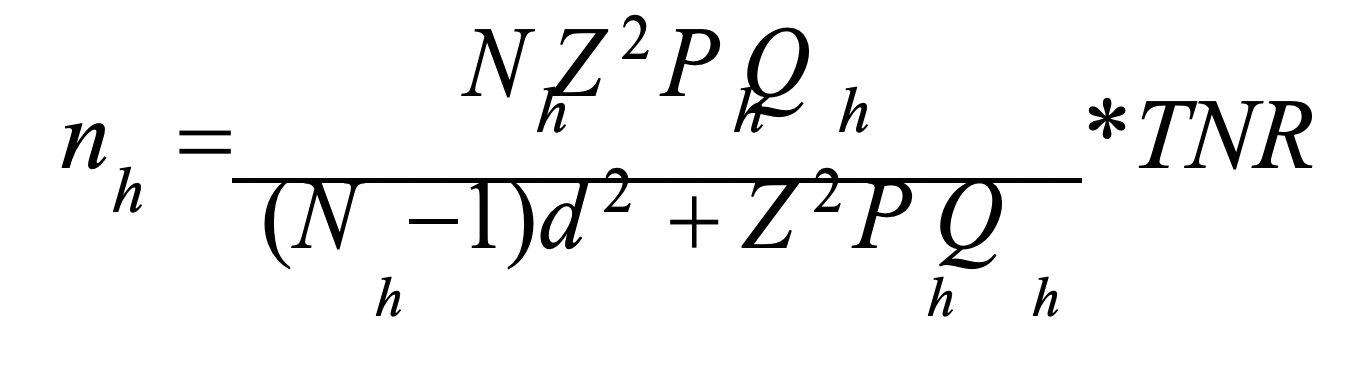


Where:

N_h_: Number of people within an age group in stratum “h”.

n_h_: Number of people within an age group in the sample in stratum “h”.

d: Margin of error assumed in the estimation of P_h_.

Z: 95% confidence interval.

TNR: Expected refusal rate due to framing or interviewing problems.

P_h_: Prevalence of overweight in adults in stratum “h”.

Q_h_: 1 - P_h_ in stratum “h”.
