# Supplementary material for: Consumption of fruits and vegetables among Peruvian adults: Analysis of a national health survey 2017–2018
Source: PLOS Glob Public Health. 2025 Mar 13;5(3):e0004222. doi: 10.1371/journal.pgph.0004222 (PMC11906043; doi:10.1371/journal.pgph.0004222)
Supplement: S2 Fig — (DOCX) [file pgph.0004222.s002.docx]

# **S2 Fig. Flowchart of data cleaning and inclusion criteria**

Total sample found in the dataset = 1091 participants (Refusal rate: 10.3%)

Sample = 914 participants

[with 24-hour recall data]

Sample = 913 participants

[with ponderation data]

Final sample size included in the analysis = 913 participants

[84% of the total sample]

Calculated sample size = 1,211 participants

N= 120 refused to participate

N= 1 without ponderation data

N= 177 without 24-hour recall data
